# Deadpan Contributes to the Robustness of the Notch Response

**DOI:** 10.1371/journal.pone.0075632

**Published:** 2013-09-24

**Authors:** A. Burcu Babaoğlan, Ben E. Housden, Marc Furriols, Sarah J. Bray

**Affiliations:** Department of Physiology, Development and Neuroscience, University of Cambridge, Cambridge, United Kingdom; University College London, United Kingdom

## Abstract

Notch signaling regulates many fundamental events including lateral inhibition and boundary formation to generate very reproducible patterns in developing tissues. Its targets include genes of the bHLH *hairy* and *Enhancer of split* [*E(spl)*] family, which contribute to many of these developmental decisions. One member of this family in *Drosophila*, *deadpan (dpn)*, was originally found to have functions independent of Notch in promoting neural development. Employing genome-wide chromatin-immunoprecipitation we have identified several Notch responsive enhancers in *dpn*, demonstrating its direct regulation by Notch in a range of contexts including the *Drosophila* wing and eye. *dpn* expression largely overlaps that of several *E(spl)* genes and the combined knock-down leads to more severe phenotypes than either alone. In addition, Dpn contributes to the establishment of Cut expression at the wing dorsal-ventral (D/V) boundary; in its absence Cut expression is delayed. Furthermore, over-expression of Dpn inhibits expression from *E(spl)* gene enhancers, but not vice versa, suggesting that *dpn* contributes to a feed-back mechanism that limits *E(spl)* gene expression following Notch activation. Thus the combined actions of *dpn* and *E(spl)* appear to provide a mechanism that confers an initial rapid output from Notch activity which becomes self-limited via feedback between the targets.

## Introduction

Notch signaling is a well-conserved pathway across metazoans involved in the regulation of many fundamental events including lateral inhibition and boundary formation. For example, in the *Drosophila* wing, activity of Notch is essential for restricting the width of the veins, the strut-like structures that confer rigidity, and for generating and maintaining the stable boundary between the dorsal and ventral cells, which acts as an organizer for patterning and growth of the whole wing. There the functions of Notch are implemented in part through the activities of *wingless* (*wg*) and *cut* (*ct*), which are expressed in a stripe at the D/V boundary in response to Notch activity.

Simple at first glance, Notch signaling is initiated by the activation of the Notch single-pass transmembrane receptor by the ligand Delta (Dl) or Serrate (Ser), which leads to its proteolysis and the release of the intracellular domain (NICD). NICD then enters the nucleus and affects transcriptional regulation by directly interacting with DNA-binding proteins of the CSL family [mammalian CBF1, *Drosophila* Suppressor of Hairless (Su(H)) and *C.elagans* LAG-1] [1], [2]. The downstream targets involved in implementing these actions include genes of the bHLH Hairy and Enhancer of split (HES) gene family, which in *Drosophila* are located within the 60 kb E(spl) complex. However, activity of the known targets cannot account for all of the functions of Notch, prompting us to perform genome wide chromatin immunoprecipitation (ChIP with anti-Su(H) antibody) to identify other Notch regulated genes [3], [4]. One gene which emerged from these analyses was *deadpan (dpn)*, itself a member of the Hairy/Enhancer of Split (HES) subclass of repressor basic Helix-Loop-Helix (bHLH) proteins, but one that had been primarily linked to the specification of neural cells. More recently it has become evident that *dpn* is Notch regulated in some contexts although the extent to which it is a direct target and its relevance to Notch functions have remained enigmatic [5].

Until recently, studies of *dpn* have predominantly focused on its roles during *Drosophila* neurogenesis [6], where its ability to repress transcription of specific genes in the precursor cell is important in promoting neurogenesis [7], [8]. Its expression persists in the neural stem cells, so-called neuroblasts, where its functions are partially redundant with those of the conventional *Drosophila E(spl)* genes [5], [9]. In this context, it remains unclear whether or not *dpn* is a Notch-regulated target because its expression is unaffected in conditions of compromised Notch signaling although it responds to high levels of Notch activity in the mature intermediate neural precursor cells (INPs) of type II neuroblast (NB) lineage [5], [9], [10]. Furthermore, the regulation and functional relevance of *dpn* in other developmental processes is only just starting to emerge. Fundamental questions that remain are whether *dpn* is a direct target of Notch in other contexts, outside the nervous system, and how its function relates to that of the other *E(spl)* genes.

In this study, we show that there are multiple Notch responsive enhancers (NREs) in the *dpn* genomic region, which direct Notch dependent expression in a variety of tissues. Expression of *dpn* therefore is a widespread response to Notch activation. In addition, we demonstrate that it has a role in regulating the establishment of stable expression of *cut* at the D/V boundary in the wing discs and that it functions in a unidirectional feedback loop with the *E(spl)* genes, helping to self limit the Notch response.

## Results

### Multiple Notch Responsive Enhancers Associated with *dpn*

**Figure 1:**
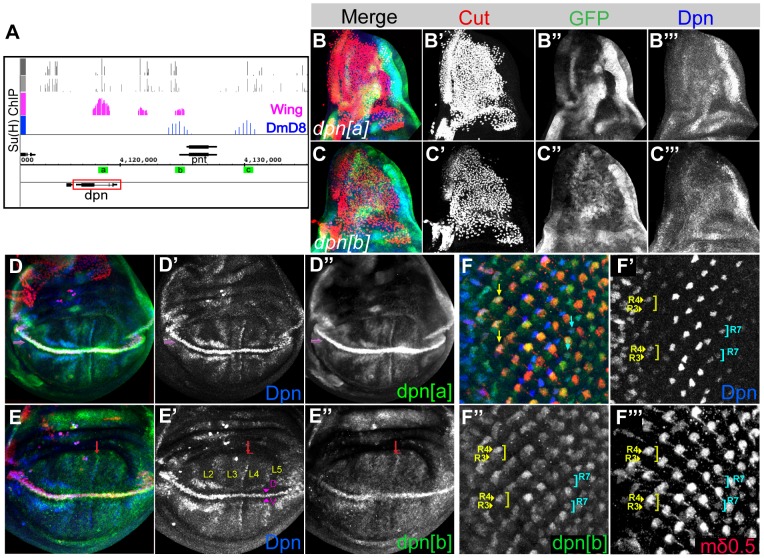
Several Notch-regulated enhancers associated with *dpn* gene. (**A**) Su(H) bound genomic regions obtained by chromatin immunoprecipitation (ChIP) using wing (pink) and DmD8 (blue) cells show strong overlap with Su(H) binding motifs (all motifs, light grey; conserved motifs, dark grey). Green a, b and c boxes represent peaks that are cloned into a reporter construct expressing GFP. (**B–C**) Thorax region of wing discs showing expression from *dpn[a]GFP* (B, green; B″ single channel) and *dpn[b]GFP* (C, green; C″ single channel) in relation to the adult muscle precursors (AMPs; red nuclei in B,C) which have similar characteristics to DmD8 cells. *dpn[b]GFP (C′′)* and endogenous Dpn (blue, single channel B′′′, C′′′) expression is detected in some of the AMPs. (**D–E)** Third instar wing discs immunostained with anti-Dpn (Blue, D, E; single channels D′, E′), anti-GFP (green D,E; single channel, D′′, E′′) and anti-Cut (red, D,E) antibodies. Both the *dpn* reporters *[a]/[b]* overlap with Dpn and Cut expression at the D/V boundary (purple arrows); *dpn[a]* also fully recapitulates Dpn expression in the interveins D-D″), whereas *dpn[b]* directs weak expression in those regions (e.g. red arrow; E-E″). (**F**) Expression of endogeneous Dpn (F′), and the *dpn[b]* reporter (F′′) overlap with *E(spl)mδ0.5LacZ* (F′′′), a Notch responsive enhancer, in third instar eye discs. Yellow and blue arrows mark the R4 and R7 cells, respectively.

*dpn* was originally reported as a pan-neural gene [7] with little evidence to suggest that it could be regulated by Notch despite the fact that it is a member of the HES family. More recently it has emerged that there is direct input of Notch into a neuroblast enhancer, although the relevance of this regulation remains unclear [5]. *dpn* expression is also detected in a wide range of other tissues including the wing, eye and leg imaginal discs, where it overlaps with well-characterised Notch target genes ([7], [11], [12] and **Fig. 1**). Whether or not Dpn is directly regulated by Notch in these tissues has remained unclear, as the characterized neuroblast enhancer does not direct expression in the imaginal discs. However, our genome-wide analysis of Notch targets has indicated that *dpn* is likely to be directly regulated by Notch in other tissues besides the central nervous system (CNS) since we detect both Su(H) binding (by chromatin immunoprecipitation; ChIP) and changes in mRNA expression in several cells and tissues including wing imaginal discs (4.6 fold upregulated after Notch activation) and DmD8 cells (muscle progenitor related; 4.8-fold upregulated after Notch activation) (**Fig. 1A**).

Depending on the cellular origin, different locations for Su(H) binding at the *dpn* locus were detected, suggesting the existence of multiple Notch responsive enhancers. The major bound region in wing imaginal discs (*dpn[a]*) was located within the second intron of *dpn*, overlapping a pair of conserved Su(H) binding motifs. Of the two other more minor Su(H) bound regions detected in wing discs, one (*dpn[b]*) coincided with a region that was also occupied in DmD8 cells and that overlapped with the neuroblast enhancer identified previously [5]. A further prominent Su(H) bound region (*dpn[c]*) was detected in DmD8 cells but flanked the adjacent *peanut* gene, so its relationship to *dpn* was less clear. All the bound regions contained sequences that have good match to Su(H) binding motifs (**Fig. 1A**; grey bar graphs).

To assess whether these Su(H) occupied regions identify Notch regulated enhancers we inserted genomic fragments encompassing the bound regions upstream of reporter genes and assessed whether they directed *dpn*-like expression patterns. First, consistent with the results from the ChIP, *dpn[b]GFP*, but not *dpn[a]GFP* (**Fig. 1B**′′** and **C′′****), recapitulated the endogenous Dpn (**Fig. 1B** **′′′** and **C′′′**) expression in the adult muscle precursors (AMPs), which have similar characteristics to DmD8 cells. Second, *dpn[a]GFP* and *dpn[b]GFP* recapitulated key aspects of *dpn* expression in other tissues. *dpn[a]*, the intronic enhancer bound by Su(H) in chromatin from wing discs, directed strong expression at the D/V margin and intervein regions, similar to endogenous *dpn* expression (**Fig. 1D**). Expression was also detected in the optic lobes of the brain, the leg joints and cone/support cells in the eye discs but not in neuroblasts or photoreceptors (**Fig. S1**). In contrast, *dpn[b]* gave weak but detectable expression at the D/V boundary and interveins in the wing, but it exhibited much stronger activity in the eye discs where it was detected in R3/R4 and R7 photoreceptors like endogenous Dpn, and was also strongly expressed in brain and leg discs (**Fig. 1E′′**, **F′′** and **Fig. S1**). *dpn[c]* did not show any significant expression in any of the tissues analyzed so its function remains unclear (Data not shown). These experiments demonstrate therefore that two of the Su(H) bound regions faithfully recapitulate endogenous *dpn* expression in specific tissues. Furthermore, their expression patterns resemble those of several *E(spl)* genes, which highlight known sites of Notch pathway activity, such as the D/V boundary and interveins in the wing as well as R4 and R7 photoreceptors in the eye [13], [14]. However, there are also some notable differences. For example, neither endogenous *dpn* nor the *dpn* enhancers exhibit strong expression in proneural territories unlike *E(spl)m7* and *E(spl)m8* [13]. In this respect the *dpn* expression appears most similar to *E(spl)mβ*, which is detected at low levels broadly through the thoracic proneural regions.

**Figure 2:**
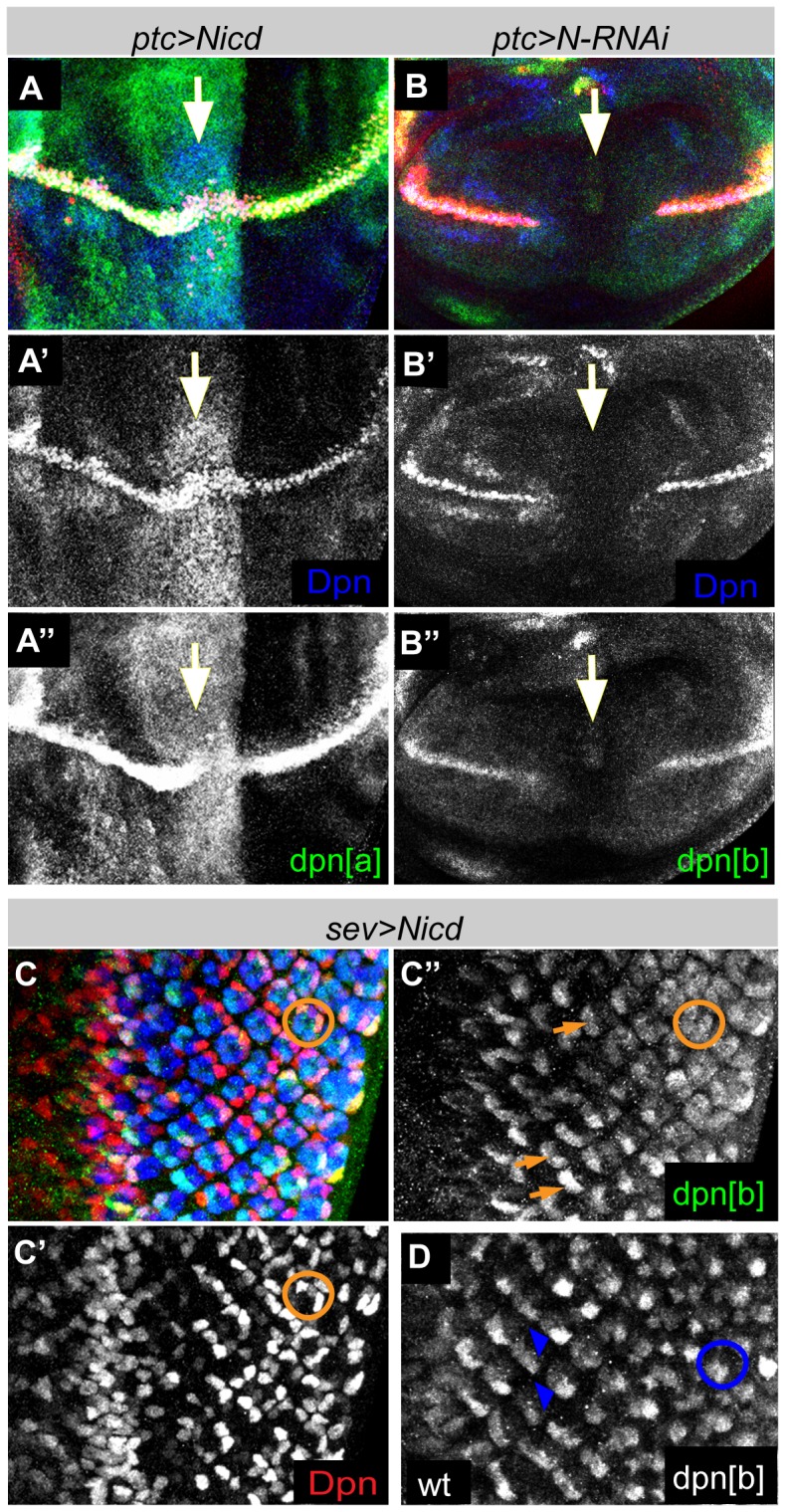
*dpn[a]* and *dpn[b]* respond to Notch. (**A–B**) Expression driven by *dpn[a]* or *dpn[b]* in third instar wing discs detected by immunostaining with anti-GFP (green) and anti-Dpn (blue). (**A-A′′**) Ectopic expression is detected when Notch intracellular domain (NICD) is expressed in the wing using *patched-Gal4* (*ptc>UAS-NICD*) (position of *ptc*-expressing region indicated by white arrow). (**B-B′′**) Downregulation of Notch via RNAi using the same driver results in ablation of both endogenous (blue) and reporter (green) expressions at the D/V boundary (white arrow). (**C-C′′**) Ectopic expression of the NICD in R1, R3, R4, R6, and R7 with *sev-Gal4* results in ectopic expression of endogenous Dpn (C′-red. Orange circle) and the GFP reporter (C′′-green. Orange circle and arrows) compared to wild type (**D**, blue circles and arrowheads).

To address whether *dpn[a]* and *dpn[b]* respond to Notch activity, we tested the consequences of manipulating Notch activity in wing and eye imaginal discs. First, expression of NICD in the wing (driven by *patched*-*Gal4* (*ptc*-*Gal4*)) was sufficient to elicit ectopic expression of *dpn[a]* and *dpn[b]*, resembling the response of the endogenous *dpn* gene **(** **Fig. 2A** and data not shown). Conversely, when Notch was down-regulated by RNAi the expression of *dpn[a] and dpn[b]* were ablated along with that of *dpn* itself (**Fig. 2B**, and data not shown). Similar results were obtained with *dpn[b]* enhancer in the developing eye. NICD driven by *sevenless*-*Gal4* (*sev*-*Gal4*, driving expression at R1, R3, R4, R6, and R7) resulted in ectopic *dpn[b]* expression so that firstly similar levels were detected in R3 and R4 (orange arrows, **Fig. 2C″**; compare to wild-type, blue arrows, **Fig. 2D**) and secondly other photoreceptor cells started to express the reporter (orange circle, **Fig. 2C″**; compare to wild-type, blue circle, **Fig. 2D**) correlating with the ectopic Dpn that was also detected (orange circle, **Fig. 2C** **′**). Together these data demonstrate that *dpn[a]* and *dpn[b]* can mediate the Notch regulation of *dpn* in the wing, while only *dpn[b]* responds in photoreceptors. We note that the *dpn[b]* fragment is larger than the previous neuroblast enhancer that was identified in this region, containing additional Su(H) sites and flanking sequences that confer additional aspects of Notch regulated expression.

#### Relationship between dpn and E(spl) gene functions

**Figure 3:**
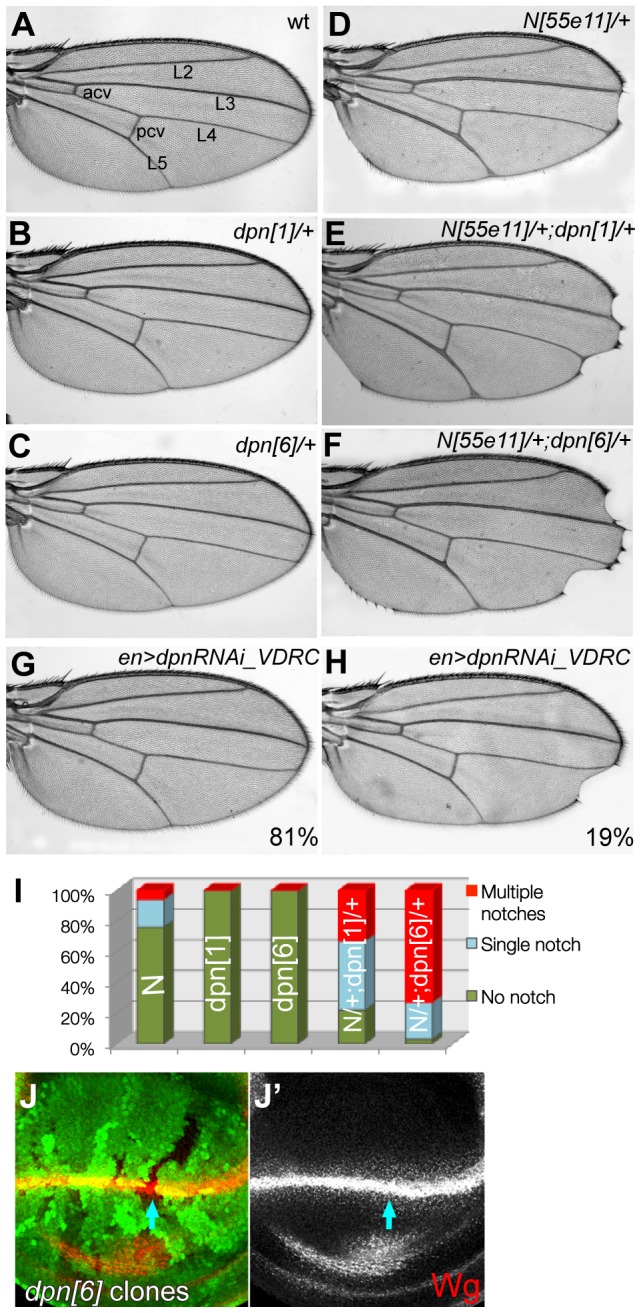
*dpn* modifies *Notch* phenotypes and directly alters the expression of the Notch target *cut*. (**A**) Wild type wing showing the wing margin and the veins: anterior cross vein (acv), posterior cross vein (pcv), L2, L3, L4 and L5. (**B–F**) *Notch* (*N[55e11]/+*) heterozygous phenotype of mild distal wing notches (D) is genetically modified in flies also heterozygous for *dpn* alleles, *dpn*[1] (B,E) and *dpn*[6] (C, F). (**G–H**) *dpn-RNAi* driven by *engrailed-Gal4 TubGal80 (en>)* caused single nicks in 19% of the wings (H). See Table S1. (**I**) Quantification of nick occurrences in each phenotype, *dpn* alleles significantly enhance the wing notching of Notch heterozygotes (p<<0.01). (**J-J′**) *dpn*[6] homozygous mutant clones do not alter Wingless (Wg) expression at the D/V boundary.

The expression of *dpn* in response to Notch activity, and the evidence showing direct binding of Su(H) in several contexts, supports the recent results indicating that *dpn* has a role in implementing Notch function in regulating proliferation [12]. This is exemplified by the fact that knock-down of *dpn* enhances the wing phenotype seen in Notch heterozygotes (**Fig 3A–F**, **Table S1** and see [12]). Twenty percent of the wings from *N[55e11]/+* female flies had a single nick in the margin at the distal tip (**Fig. 3D**). In combination with *dpn* alleles both the extent and the frequency of wing nicks were enhanced. Thus, 97% of *N[55e11]/+*; *dpn*[6]*/+* wings contained wing nicks and these frequently extended into the posterior of the wing margin (**Fig. 3F**). Slightly lower frequency was detected in combinations with *dpn*[1] where 78% of wings contained nicks, but again this was significantly enhanced compared to the *N[55e11]/+* alone (**Fig. 3E** comparing to **3D**). In contrast, reductions in *E(spl)* function did not enhance the wing notching phenotype of *Notch* heterozygotes under these conditions (**Table S1**). These genetic interactions therefore support the hypothesis that *dpn* specifically contributes to Notch function at the D/V boundary in the wing.

**Figure 4:**
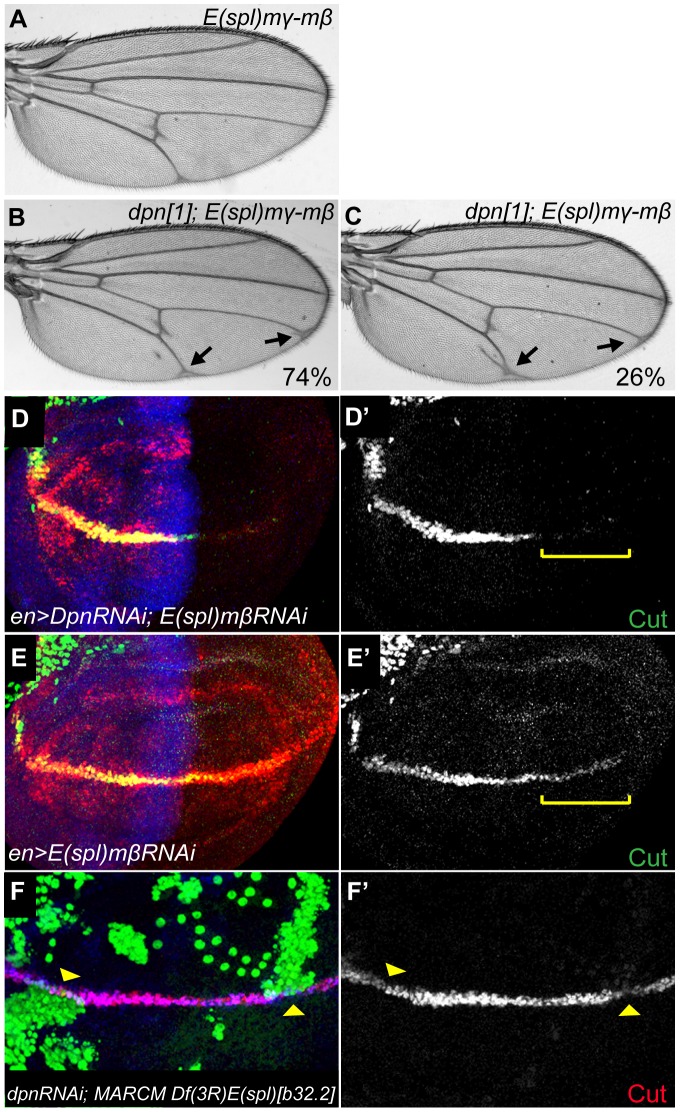
*dpn* and *E(spl)* genes may act redundantly. (**A–C**) The mild wing vein phenotype of *E(spl)mγ-mβ* (A) is dominantly modified by *dpn*[1] (B and C). (**B**) Arrows point to vein thickening at the tips of L4 and L5, observed in 74% of *dpn*[1]*/+ E(spl)mγ-mβ* wings (**C**) 26% of *dpn*[1]*/+ E(spl)mγ-mβ* wings also showed ectopic veins surrounding L5 (arrow). See Table S2. (**D–E**) *E(spl)*m*β* (E-E′) or both *dpn* and *E(spl)*m*β* (D-D′) levels were knocked down by RNAi at early third instar wing discs. Cut expression (green) was dramatically reduced in the double RNAi (D′) compared to *E(spl)*m*β*-*RNAi* alone (E′). (**F-F′**) *E(spl)-C* loss of function MARCM clones produce reduction in *Cut* expression (F′) when combined with *dpn RNAi*.

Despite the genetic interactions with *Notch*, remarkably few defects were detected in *dpn* mutant clones or in tissue expressing RNAi targeting *dpn* (e.g. **Fig 3J** and **Fig 4**). For example, the majority of adult flies showed no defects under conditions where *dpn-RNAi* was expressed throughout the posterior of the wing using *engrailed-Gal4*. At best there was a low penetrance of wing nicks (19%) with one RNAi line, consistent with *dpn* having a subtle role in this process (**Fig. 3H**). Similar lack of phenotype was seen when *E(spl)* function was eliminated using a deficiency that spans the entire locus [15]. One possibility, given their shared Notch regulation, is that these HES genes have related functions as recently suggested [9], [12], so that the combined activity is required for the robust establishment of Notch function. Elimination of all the *E(spl)* genes causes embryonic lethality [15]. However, a newly generated deficiency, *E(spl)mγ-mβ [DK33]*, that removes only two *E(spl)bHLH* genes (*E(spl)mγ* and *E(spl)mβ*) has a mild wing vein phenotype (**Fig. 4A**). We therefore assessed whether this phenotype could be modified by a reduction in the levels of *dpn*, by making combinations with heterozygous *dpn* alleles (**Fig. 4A–C**,**Table S2**). Wings from homozygous *E(spl)mγ-mβ [DK33]* flies had mild thickening in the posterior cross-vein (pcv) and tip of L5 (**Fig. 4A**). This mild phenotype was dominantly enhanced by removing a single copy of *dpn*, giving rise to prominent vein thickening at the tips of L4 and L5 (**Fig. 4B**) as well as to ectopic veins flanking L5 in 26% of wings (**Fig. 4C**). This genetic interaction suggests that *dpn* and *E(spl)* function together during wing vein development.

As there are few specific Notch targets known in the developing veins, we extended our analysis to Notch function at the D/V boundary in the wing, where there are several known targets of Notch activity including *ct*. In larvae, combined knock-down of *E(spl)mβ* and *dpn* expression in the posterior compartment of the third instar wing disc led to significant reduction of Cut level in comparison to *E(spl)mβ* alone, although we note that the latter caused a subtle decrease (compare **Fig. 4D-D** **′** to **4E-E′**). To further assess whether *dpn* made a contribution to the regulation of *ct* expression, *dpn* function was eliminated in clones of cells that completely lacked all *E(spl)* functions using a deletion of the complex, *Df(3R)E(spl)[b32.2]*, which has been combined with a *gro*+ transgene to rescue any effects on the neighbouring *gro* gene [15]. Such *dpnRNAi*; *Df(3R)E(spl)[b32.2]* double mutant clones had lost Cut staining, although knock down of either alone failed to significantly compromise *ct* expression under these conditions (**Fig 4F**;data not shown). Similar analysis in the eye disc also suggested that the genes have related functions. Knock down of *dpn* alone produced few if any defects (some rotational defects but no fate changes observed) while knock down of *dpn* and *E(spl)mδ* resulted in rare examples of symmetrical ommatidia (it was not possible to test the combinations with *Df(3R)E(spl)[b32.2]* as this leads to early defects in patterning; data not shown). Thus these data suggest that the *dpn* and *E(spl)* genes have overlapping functions in eye and wing development.

**Figure 5:**
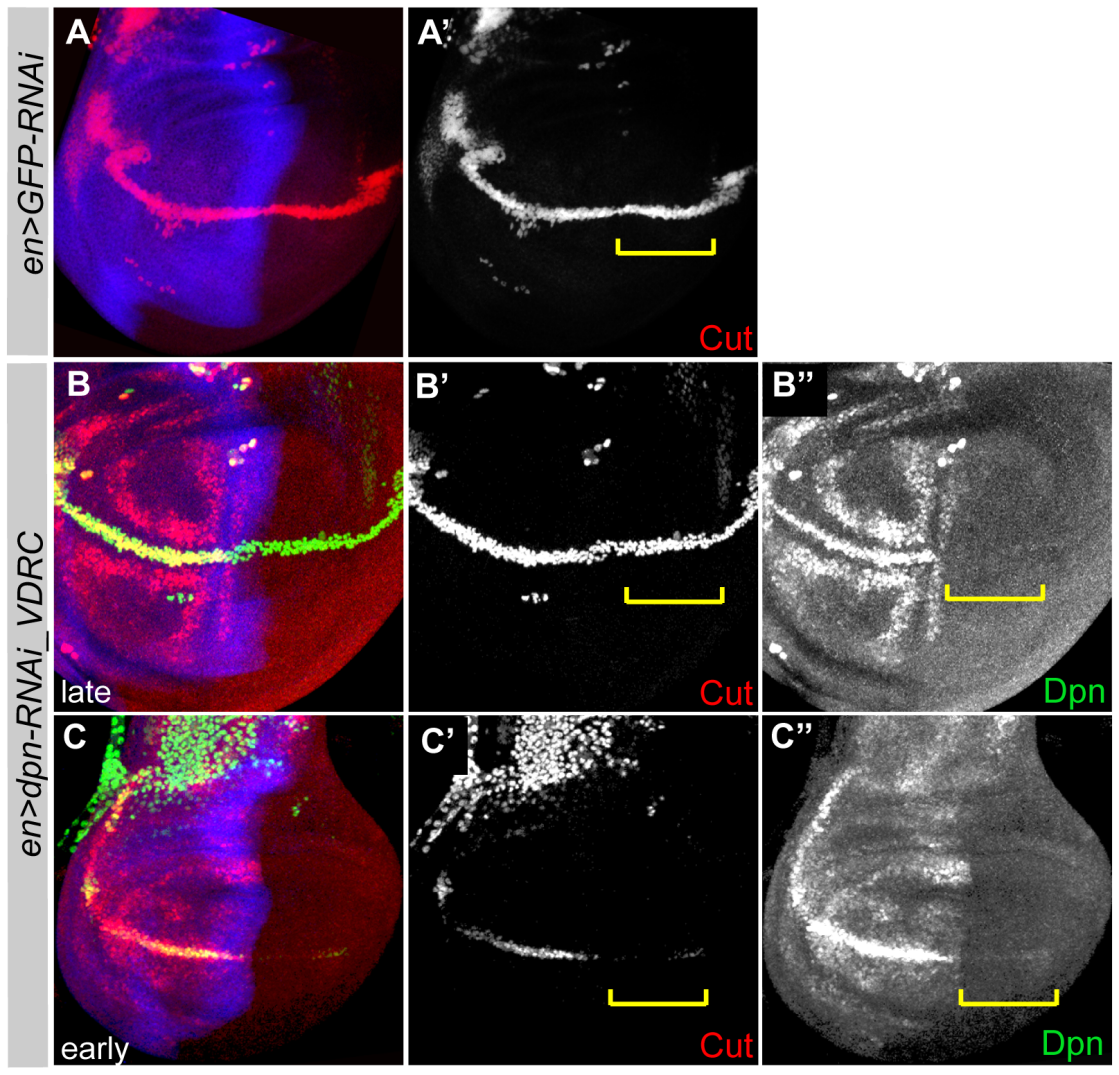
*dpn* regulates *Cut* expression. *dpn* downregulation, mediated by *en>RNAi*, in the posterior compartment of late (B-B′′) and early (C-C′′) third instar wing discs. *GFP-RNAi* was used as a control (A-A′). Discs were stained with Cut (red) and Dpn (green). Yellow brackets (B′-B′′ and C′-C′′) represent the Cut expression at the posterior D/V boundary.

One surprising result emerged when we analyzed the consequences of ablating *dpn* in early wing discs (**Fig. 5**). We found that the RNAi mediated knock down of *dpn* in the posterior compartment of the wing resulted in down-regulation of Cut at the D/V margin in mid third instar discs but not in older discs (**Fig. 5C**). Similar results were seen using independent RNAi lines, all produced defects at early but not at later stages (**Fig. S2**). Together the results suggest that *dpn* may make a contribution to Notch function at the D/V boundary, but that its absence can be compensated by another mechanism, to explain the recovery of *cut* expression at later stages, and the lack of phenotype in the adult wings. It is also striking that the phenotype is one of reduced Cut expression, because *dpn* and the *E(spl)bHLH* genes have characteristics of transcriptional repressors. This suggests that there may be an intermediary factor that is repressed by Dpn to enable the expression of Cut (see discussion). We therefore drew up a list of candidates, that are expressed in the relevant domain of the wing disc, and tested whether they were inhibited by Dpn overexpression or up-regulated in conditions of *dpn-RNAi*. None of those tested (e.g. *msh*, *dve*, others) showed any discernable change in response to changes in *dpn* (**Fig. S3**).

#### Regulation of E(spl)bHLH genes by dpn

**Figure 6:**
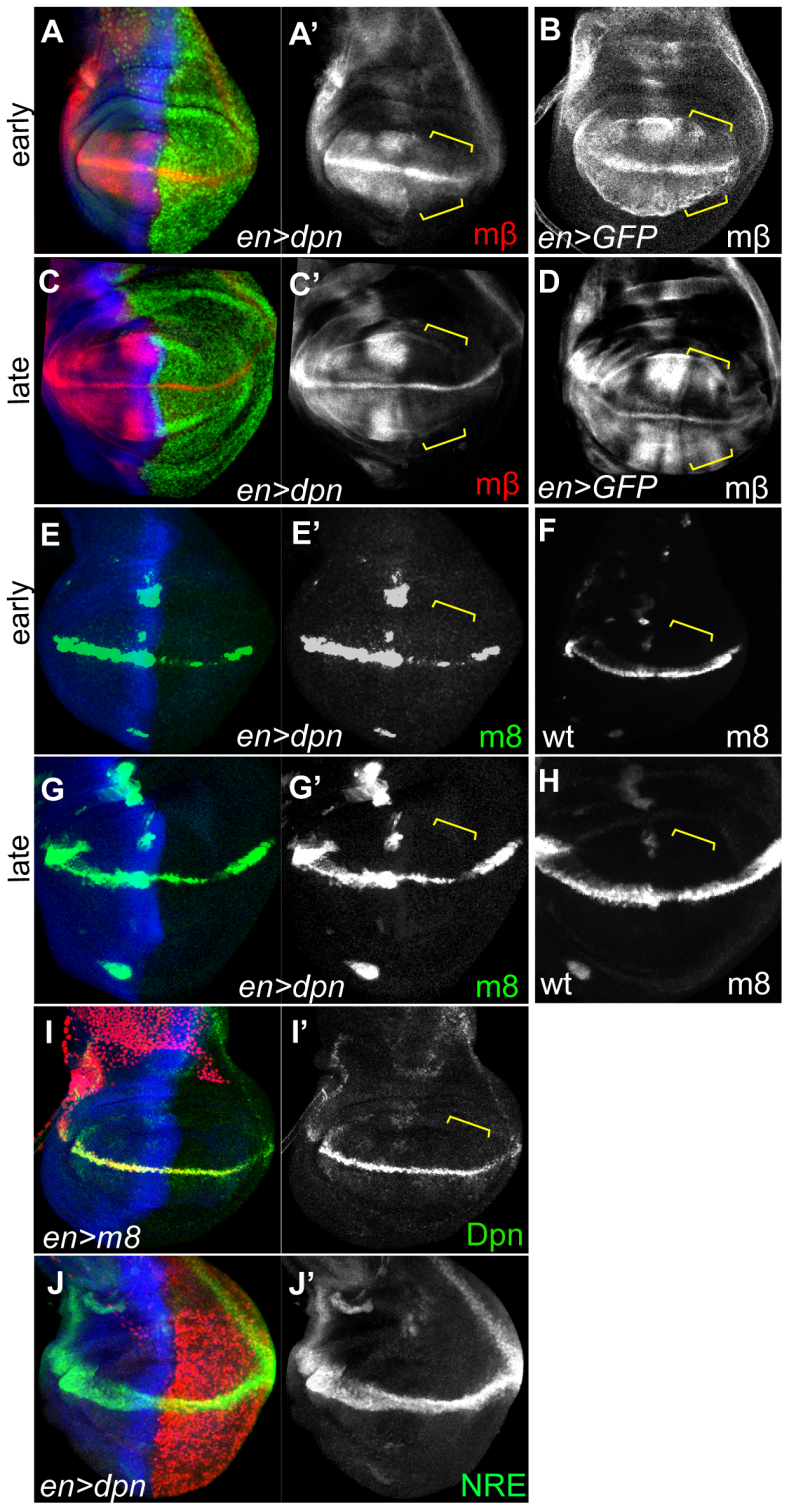
*dpn* regulates *E(spl)* genes. Early (**A, B, E and F**) and late (**C, D, G and H**) third instar wing discs with *en*> driving *dpn* expression. Yellow brackets highlight the posterior compartment in which engrailed is expressed. *E(spl)mβ* (A′, C′) and *E(spl)m8-lacZ* (E′, G′) expression were reduced in both early and late instar discs, in comparison to GFP overexpression (B and D) and wild type *E(spl)m8-lacZ* (F and H) controls. (**I**) Misexpression of *E(spl)m8* fails to alter Dpn expression. (**J**) Misexpression of *dpn* has no effect on *NRE-GFP*.

*dpn* and *E(spl)bHLH genes* are all regulated by Notch and have broadly similar expression patterns. This raises the possibility that there could be cross-regulatory interactions between these different HES genes, similar to what has been observed in somitogenesis [16]. We therefore tested whether ectopic expression of *dpn* or of *E(spl)bHLH* genes was able to modify the other's expression. First we found that overexpression of *dpn* (using *enGal4 tubGal80ts)*, but not a control GFP, was sufficient to suppress the expression of two *E(spl)* reporters, *E(spl)mβ1.5-CD2 and E(spl)m8-lacZ* (**Figure 6A–H**). This reduction was even detected in late stage wing discs (**Fig 6C and 6G**). As there was no effect on a generic Notch reporter, *NRE-GFP* [17], under equivalent conditions (**Fig 6J-J** **′**), it is likely that *dpn* regulates the *E(spl)* enhancers directly rather than acting indirectly through Notch. In contrast, over-expression of *E(spl)m8*, *E(spl)mβ*, *E(spl)m7* or *E(spl)m5* (**Fig. 6I**, **Fig S4**) all failed to modify the *dpn* expression at the D/V boundary. The data suggest therefore that there is a unidirectional feedback loop between Notch targeted HES genes, with *dpn* regulating *E(spl)bHLH* and not *vice versa*.

## Discussion

HES genes are well-known targets of Notch activity. However, in *Drosophila* only the bHLH genes within the *E(spl)* Complex were originally thought to be directly downstream of *Notch*. The expression of another *HES* genes, *dpn*, appeared independent of Notch and indeed was associated with cells where Notch activity is considered to be down-regulated (embryonic neuroblasts). More recently it has emerged that *dpn* expression is under Notch regulation in some contexts [5], [9]. Our results extend these findings by demonstrating that *dpn* is directly bound by Su(H) *in vivo*. As the Su(H) occupied regions differ according to the tissue-type, it appears that *dpn* contains several Notch responsive enhancers and our results demonstrate that these direct Notch-dependent expression in different subsets of tissues. Nevertheless, it is striking that a single *dpn* enhancer, *dpn[b]* exhibits Notch related expression in both the eye and the wing discs yet these patterns are characteristic of distinct genes/enhancers from the *E(spl)* Complex [13].

Despite the clear regulation by Notch, there is however relatively few phenotypes resulting from loss of *dpn* in many tissues. For example, both the wing and eye disc exhibit robust expression of *dpn* but neither exhibit phenotypes when *dpn* function was ablated. However, genetic interactions demonstrate that *dpn* function is related to Notch and both our evidence, and that from recent studies [9], [12], indicate that it has partially redundant functions with the *E(spl)* genes. This is exemplified by the fact that absence of *dpn* or of *E(spl)* Complex alone has little effect on the D/V boundary, but the combined knock down leads to loss of key gene expression.

**Figure 7:**
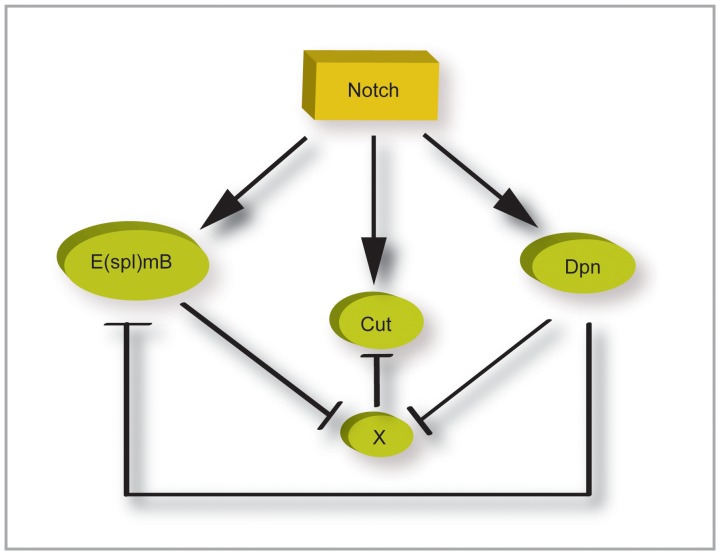
*dpn* contributes to the robustness of the Notch response. Diagram summarizing regulatory interactions between the genes indicated. Involvement of additional gene, X, is inferred due to the fact that HES genes appear to function as dedicated repressors.

What then is the relevance of *dpn* in these contexts, especially given that there are 7 *E(spl)bHLH* genes that also appear to have largely redundant functions? We propose two components to *dpn* function to explain its importance in the Notch response. Clues for the first come from the fact that we could detect subtle phenotypes from reductions in *dpn* when we analyzed early developmental stages. Thus the absence of *dpn* led to a delay in the ability of Notch to up-regulate *cut*. Earlier studies also demonstrated a subtle decrease in *cut* expression in cells lacking *E(spl)* genes [18]. These results could be explained if both *E(spl)* and *dpn* make a contribution to *cut* regulation. We suggest that this must be indirect, via the inhibition of a repressor, since both *dpn* and *E(spl)bHLH* are thought to be dedicated repressors (**Fig. 7**). So far, we have not identified another repressor that could act as an intermediary.

The second component of *dpn* function is suggested by the observation that Dpn can repress the enhancers derived from *E(spl)bHLH* genes but not vice versa. Futhermore, we observed that cells with high levels of Dpn often had lower levels of E(spl)bHLH on a cell by cell level. We therefore propose that there is a direct regulatory relationship between *dpn* and *E(spl)bHLH*, whereby *dpn* represses *E(spl)bHLH* expression (**Fig. 7**). This could set a maximum threshold for *E(spl)* gene expression since, in previous studies, we have found that *dpn* shows a less rapid up-regulation following Notch activation than the *E(spl)* genes [19]. This is reminiscent of the differences seen between HES gene responses in the oscillatory clock involved in somitogenesis [20] and suggests that similar HES gene cross-regulatory network may underpin other Notch dependent processes.

## Materials and Methods

### *Drosophila* Genetics and Stocks

All alleles and stocks are described in FlyBase (www.flybase.org) unless indicated otherwise. The following mutant lines were used: *dpn* [*1*], *dpn* [*6*], *Notch[55e11]*, *E(spl)[b32.2] gro* ^+^ *FRT82B*, *E(spl)Δmγ-mβ* [DK33-10.1] (see below). The following reporters were used: *dpn[a]GFP*, *dpn[b]GFP*, *E(spl)mδ0.5*-*LacZ* [21], *E(spl)m8-LacZ* [22] *E(spl)mβ1.5-CD2* [23], *NRE-GFP* [17].

To assess the interactions between *dpn* and *Notch*, the following crosses were performed:

*Notch[55e11]/FM7* females were mated to *dpn* [*1*] */CyO* and *dpn* [*6*] */CyO* males in independent crosses. *Notch[55e11]/+*; *dpn* [*1*] */+* and *Notch[55e11]/+*; *dpn* [*6*] */+* female adult wings were dissected out and analysed in terms of the wing nicks occurrence. As controls, *Notch[55e11]/FM7*, *dpn* [*1*] */CyO* and *dpn* [*6*] */CyO* females were individually crossed to wild type males in independent crosses. *Notch[55e11]/+*, *dpn* [*1*] */+* and *dpn* [*6*] */+* adult wings were analysed individually.

In overexpression and RNAi experiments, Gal4 driver stocks (*engrailed*:*Gal4 Tub*:*Gal80^ts^*, *patched*:*Gal4 Tub*:*Gal80^ts^*, *sevenless-Gal4*) were combined with UAS lines and larvae were shifted to 30°C 48 hours after egg laying. The following RNAi and overexpression lines were used: *UAS-Notch-intra[79.2]*, *UAS-dpn* (from H.Vaessin), *UAS-E(spl)m8* [24], *UAS-dpn-RNAi* (VDRC- v106181), *UAS-Notch-RNAi* (BL-7078), *UAS-E(spl)mβ-RNAi* (BL26202), *UAS-GFP-RNAi* (BL-9330). *UAS-E(spl)m5* [18], *UAS-m5RNAi* (BL-26201), *UAS-E(spl)m7*, *UAS-dpnRNAi* (BL-26320), *UAS-dpnRNAi* (DGRC-8704R-4).

Mitotic clones were generated by FLP-mediated mitotic recombination [25]. Clones lacking *dpn* were obtained by crossing *FRT42B dpn* [*6*] */CyO* males to *hsp-FLP122; FRT42B/CyO* virgin females. Control clones were generated using the wild type *FRT42B* chromosome. The progeny of these crosses were heat shocked at 37°C for 1 h between 48 and 72 h after egg-laying. Discs were dissected and analyzed 3 days after the induction of the clones.

To generate MARCM clones [26], *E(spl)[b32.2] gro* ^+^ *FRT82B/TM6B or UAS-dpn-RNAi*; *E(spl)[b32.2] gro* ^+^ *FRT82B/TM6B* were crossed to *hs-FLP tubGal4 UAS-GFP*; *FRT82B tubGal80*. Progeny were heat shocked at 37°C for 1 hr after 72 hours egg-laying then kept at 30°C until dissection.

Generation of *E(spl)Δmγ-mβ* [DK33-10.1]:

Small deficiencies in the *E(spl)-Complex* were generated in a cross to induce P-element mediated male recombination [27] using the P-element: *P{lacW}K33* [28], where the transposon is inserted 61 bp upstream of the transcriptional start site of *E(spl)mγ*, oriented with the 3′ terminal repeats close to *E(spl)mγ*. One of the progeny had a deletion of approximately 6.2 Kb, starting inside the P-element (retaining the last 2361 bp of the 5′-end of the *P{lacW}*) deleting all the *E(spl)mγ* gene and most of *E(spl)mβ* coding region, terminating after the first 31amino acids.

For genetic interactions experiments, Notch[55e11]/FM7 females were mated to both E(spl)[b32.2] gro^+^FRT82B/TM6B and homozygous viable E(spl)Δmγ-mβ [DK33-10.1] males in independent crosses. Notch[55e11]/+; E(spl)[b32.2] gro^+^FRT82B/+ and Notch[55e11]/+; E(spl)Δmγ-mβ [DK33-10.1]/+ female adult wings were selected and analysed in terms of wing nick occurrences. The Chi-squared test was used to evaluate statistical significance.

### Molecular Cloning

Putative NREs in *dpn* region:

ChIP-enriched regions (putative NREs in *dpn*) were amplified from *Drosophila* genomic DNA using primers containing specific restriction enzyme sequences and cloned into pGreenRabbit/pRedRabbit vectors [17] for *in vivo* reporter assays. Sequences of primers and restriction enzymes used for cloning *dpn* reporters are as follows:

***dpn[a]*** chr2R- KpnI and XbaI

Left ACGTTTCGTGCCTCATATGTC

Right TTAAGGCACAAGTGTCCGAAG

***dpn[b]*** chr2R-NotI and KpnI

Left AAAACAGGAGTCGCTTTGGA

Right GCAGTGTGACCCTGGAAAAT

***dpn[c]*** chr2R-XbaI and KpnI

Left AGTGTGTGCGTGCGTAAAAG

Right CACAACAAAAGCGAACGAAA

### Immunostaining

Antibody staining of wing and eye imaginal discs was performed as described previously [21] with the following antibodies: guinea pig anti-Dpn (1∶1000; gift of J. Skeath, Washington University in St Louis, MO, USA), rat anti-Ci (1∶20; Developmental Studies Hybridoma Bank [DSHB]), mouse anti-Cut (1∶20; DSHB), mouse anti-GFP (1∶500, Invitrogen), mouse anti β-gal (1∶20; DSHB), rabbit anti-Dve [29], rabbit anti-Msh [30]. Images were acquired with a scanning confocal microscope (Leica TCS SP2) and processed using Photoshop (Adobe).

## Supporting Information

Figure S1 Reporter [a] expression was detected in the leg joints (A), the optic lobes of the brain (C), and cone/support cells in the eye discs (E) but not in neuroblasts or photoreceptors, while reporter [b] expression was detected in leg discs (B), in brain neuroblast lineages (D) and in R3–R4 and R7 photoreceptors (F).
(TIF)

Figure S2 *dpn* downregulation mediated by two different RNAi lines using the *enGal4* system at the posterior compartment of early (A–C) and late (B–D) third instar wing discs. Discs were stained with Dpn (red), Cut (green) and Ci (blue).
(TIF)

Figure S3 (A-A′) Msh expression does not change at third instar wing discs after *dpn* misexpression at the posterior compartment (marked by the absence of blue) compared to wild type (B). (C-C′) Similarly Dve expression does not respond to *dpn* misexpression at the posterior compartment (marked by the absence of red) at third instar larval stages compared to wild type (D).
(TIF)

Figure S4 Neither *E(spl)m5* misexpression (A-A′) nor *E(spl)m5-RNAi* driven by *en-Gal4* changes Dpn expression (green in A and red in B) in the wing disc. Similarly *dpn* expression stays the same after misexpression (C-C′) or RNAi (D-D′) of *E(spl)mβ*. (E-E′) *E(spl)m7* misexpression does not cause any difference in *dpn* expression.
(TIF)

Table S1 Genetic interaction between *Notch* and *dpn*.
(DOC)

Table S2 Genetic interaction between *dpn* and *E(spl)*.
(DOC)
