# Supplementary figures and images for: Deadpan Contributes to the Robustness of the Notch Response
Source: PLoS One. 2013 Sep 24;8(9):e75632. doi: 10.1371/journal.pone.0075632 (PMC3782438; doi:10.1371/journal.pone.0075632)

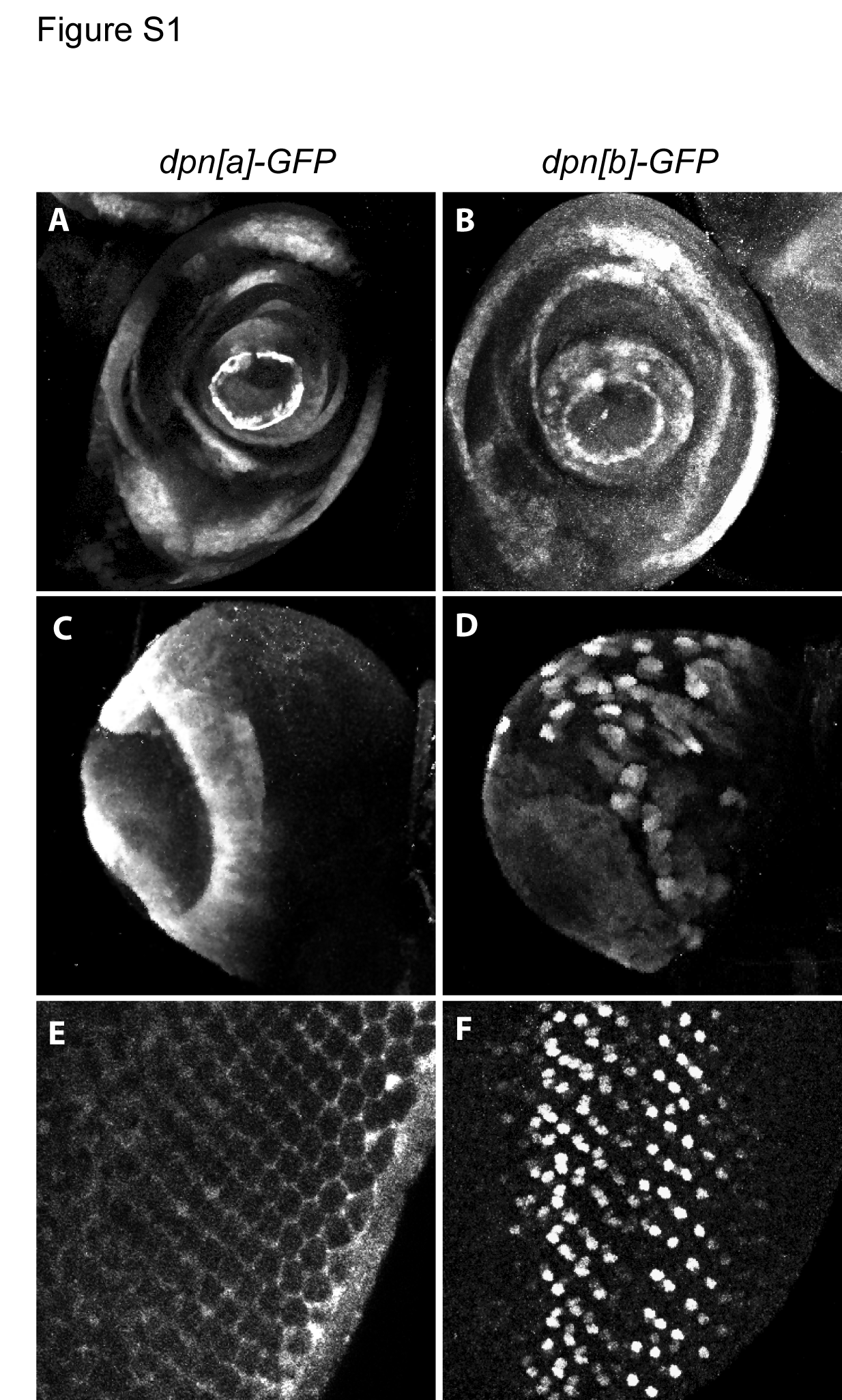

Supplement: Figure S1 — Reporter [a] expression was detected in the leg joints (A), the optic lobes of the brain (C), and cone/support cells in the eye discs (E) but not in neuroblasts or photoreceptors, while reporter [b] expression was detected in leg discs (B), in brain neuroblast lineages (D) and in R3–R4 and R7 photoreceptors (F). (TIF) [file pone.0075632.s001.tif]

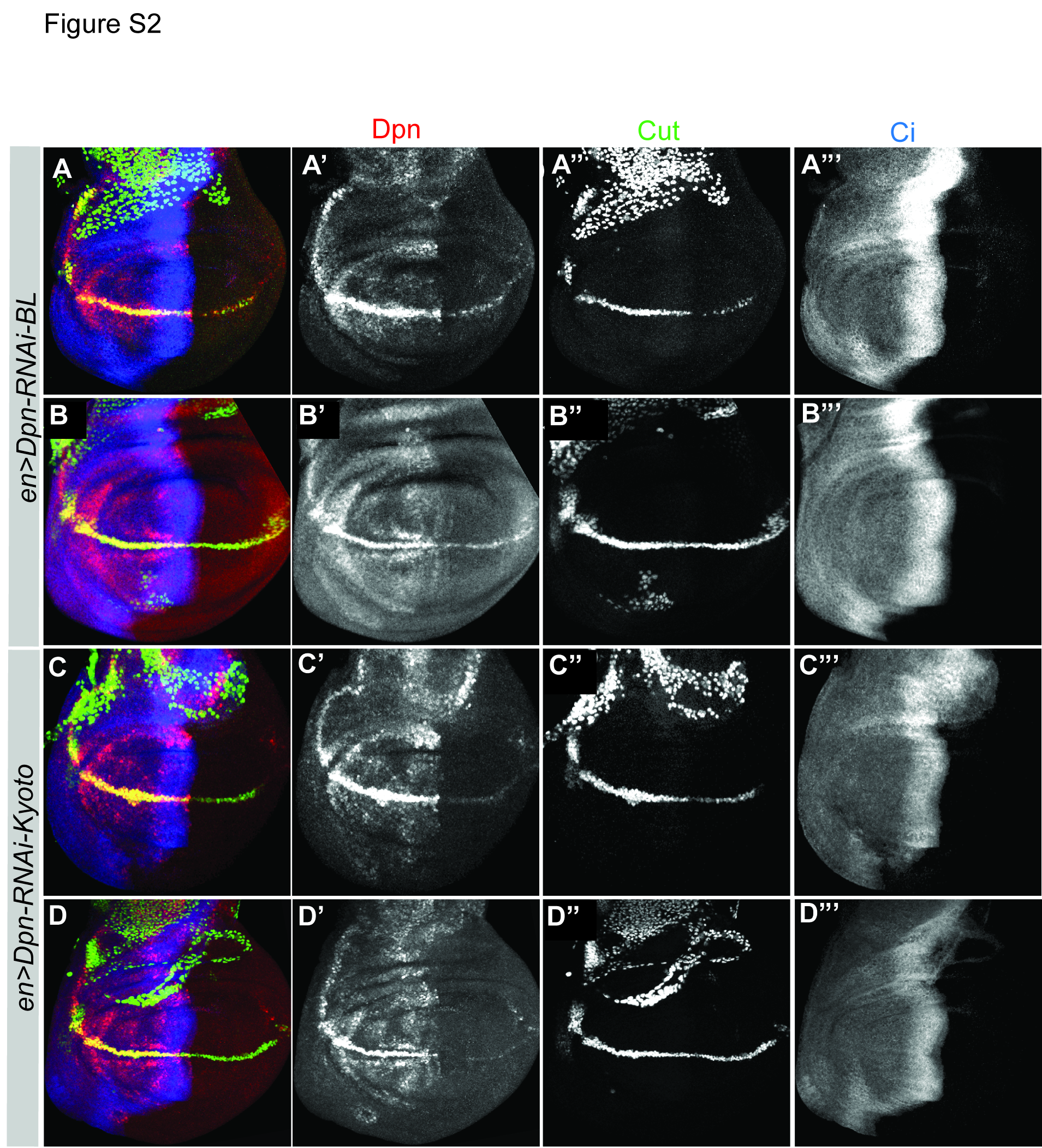

Supplement: Figure S2 — dpn downregulation mediated by two different RNAi lines using the enGal4 system at the posterior compartment of early (A–C) and late (B–D) third instar wing discs. Discs were stained with Dpn (red), Cut (green) and Ci (blue). (TIF) [file pone.0075632.s002.tif]

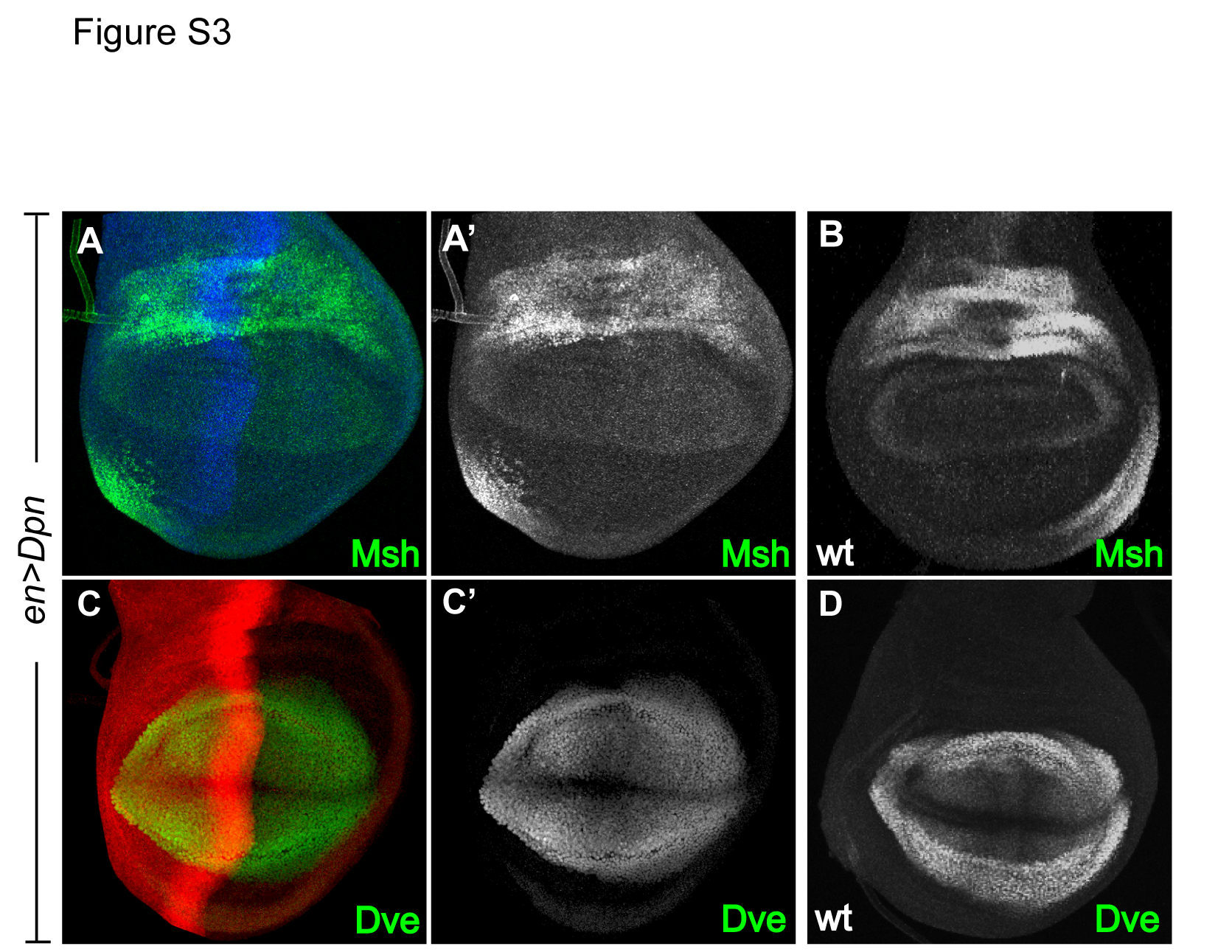

Supplement: Figure S3 — (A-A′) Msh expression does not change at third instar wing discs after dpn misexpression at the posterior compartment (marked by the absence of blue) compared to wild type (B). (C-C′) Similarly Dve expression does not respond to dpn misexpression at the posterior compartment (marked by the absence of red) at third instar larval stages compared to wild type (D). (TIF) [file pone.0075632.s003.tif]

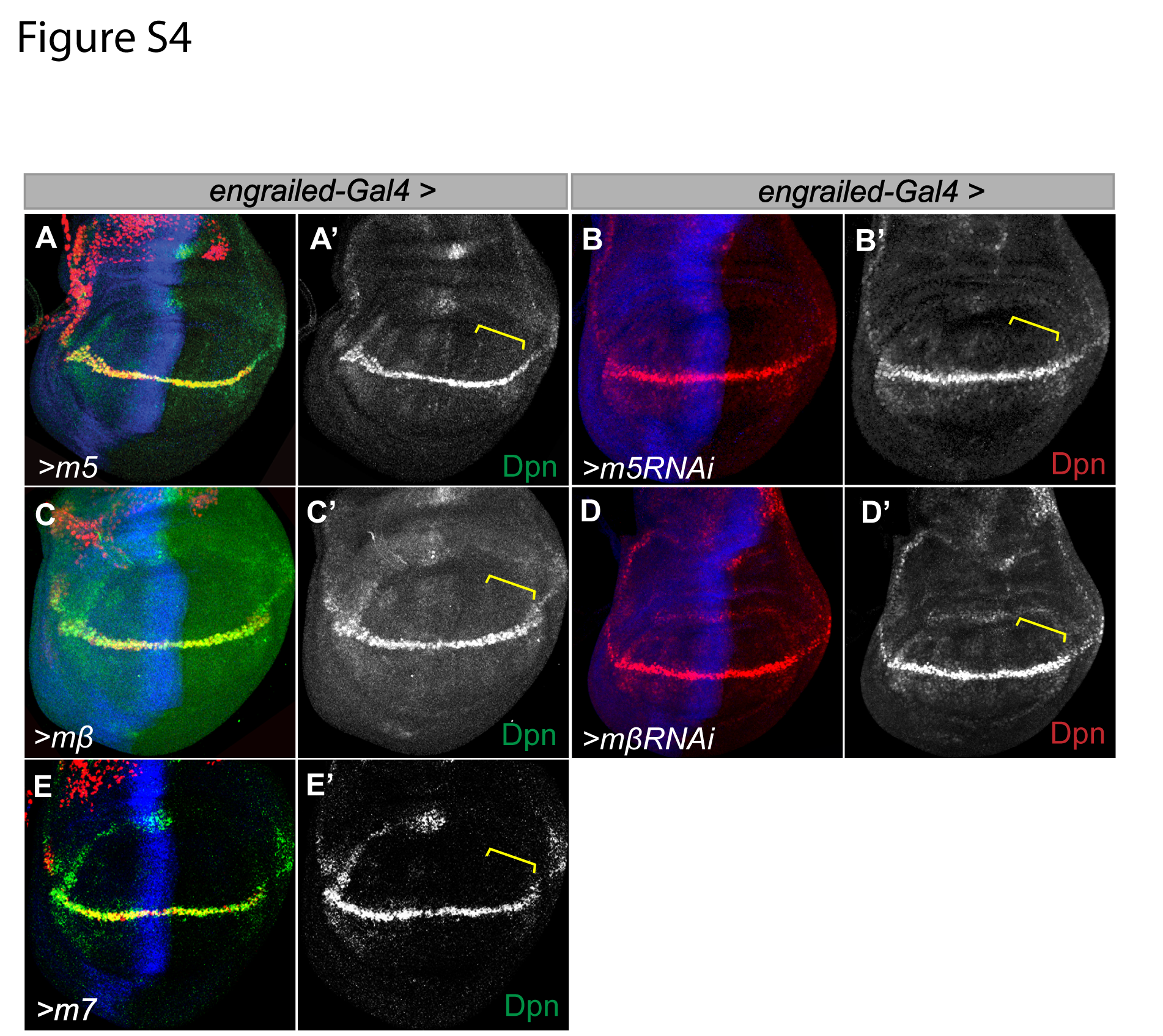

Supplement: Figure S4 — Neither E(spl)m5 misexpression (A-A′) nor E(spl)m5-RNAi driven by en-Gal4 changes Dpn expression (green in A and red in B) in the wing disc. Similarly dpn expression stays the same after misexpression (C-C′) or RNAi (D-D′) of E(spl)mβ. (E-E′) E(spl)m7 misexpression does not cause any difference in dpn expression. (TIF) [file pone.0075632.s004.tif]
