# Supplementary material for: Deadpan Contributes to the Robustness of the Notch Response
Source: PLoS One. 2013 Sep 24;8(9):e75632. doi: 10.1371/journal.pone.0075632 (PMC3782438; doi:10.1371/journal.pone.0075632)
Supplement: Table S1 — Genetic interaction between Notch and dpn. (DOC) [file pone.0075632.s005.doc]

**Table S1:** Genetic interaction between *Notch* and *dpn*

|  | No nick  (% wings) | Single nicks  (% wings) | Multiple nicks  (% wings) |
| --- | --- | --- | --- |
| *N[55e11]/+* (n=72) | 76 | 18 | 6 |
| *dpn[1]/+* (n=75) | 100 | 0 | 0 |
| *dpn[6]/+* (n=70) | 100 | 0 | 0 |
| *N[55e11]/+; dpn[1]/+* (n=119) | 22 | 45 | 33 |
| *N[55e11]/+; dpn[6]/+* (n=154) | 3 | 24 | 73 |
| *N[55e11]/+; E(spl)mγ-mβ[DK33]/+* (n=52) | 71 | 29 | 0 |
| *N[55e11]/+; Df(3R)E(spl)[b32.2]/+* (n=22) | 77 | 23 | 0 |

Quantifications obtained from independent crosses performed in parallel.

Letter “n” designates the total number of wings quantified from female flies.
