# Supplementary material for: Deadpan Contributes to the Robustness of the Notch Response
Source: PLoS One. 2013 Sep 24;8(9):e75632. doi: 10.1371/journal.pone.0075632 (PMC3782438; doi:10.1371/journal.pone.0075632)
Supplement: Table S2 — Genetic interaction between dpn and E(spl). (DOC) [file pone.0075632.s006.doc]

**Table S2:** Genetic interaction between *dpn* and *E(spl)*

|  | Ectopic vein formation at L5 (% wings) | Vein thickening at L5 (% wings) | | Vein thickening at L4 (% wings) | Vein thickening at pcv (% wings) |
| --- | --- | --- | --- | --- | --- |
|  |  | Mild Strong | |  |  |
| *dpn[1]/+* (n=75) | 0 | 0 | 0 | 0 | 0 |
| *E(spl)mγ-mβ[DK33]=* (n=52) | 0 | 100 | 0 | 0 | 100 |
| *dpn[1]/+; E(spl)mγ-mβ[DK33]=* (n=203) | 26 | 0 | 74 | 100 | 100 |

Quantifications obtained from independent crosses performed in parallel.

Letter “n” designates the total number of wings quantified from female flies.

Symbol = designates homozygous viable.
